# Elevated Plasma IL-6 Associates with Increased Risk of Advanced Fibrosis and Cholangiocarcinoma in Individuals Infected by *Opisthorchis viverrini*

**DOI:** 10.1371/journal.pntd.0001654

**Published:** 2012-05-22

**Authors:** Banchob Sripa, Bandit Thinkhamrop, Eimorn Mairiang, Thewarach Laha, Sasithorn Kaewkes, Paiboon Sithithaworn, Maria Victoria Periago, Vajarabhongsa Bhudhisawasdi, Ponlapat Yonglitthipagon, Jason Mulvenna, Paul J. Brindley, Alex Loukas, Jeffrey M. Bethony

**Affiliations:** 1 Department of Pathology, Khon Kaen University School of Medicine, Khon Kaen, Thailand; 2 Department of Epidemiology and Biostatistics, Khon Kaen University School of Public Health, Khon Kaen, Thailand; 3 Department of Radiology, Khon Kaen University School of Medicine, Khon Kaen, Thailand; 4 Department of Parasitology, Khon Kaen University School of Medicine, Khon Kaen, Thailand; 5 Instituto René Rachou, Belo Horizonte, Minas Gerais, Brazil; 6 Department of Surgery, Khon Kaen University School of Medicine, Khon Kaen, Thailand; 7 Liver Fluke and Cholangiocarcinoma Research Center, Khon Kaen University, Khon Kaen, Thailand; 8 Queensland Tropical Health Alliance, James Cook University, Cairns, Queensland, Australia; 9 Department of Microbiology, Immunology & Tropical Medicine, George Washington University Medical Center, Washington, D. C., United States of America; Hospital Universitário, Brazil

## Abstract

*Opisthorchis viverrini* is considered among the most important of the food-borne trematodes due to its strong association with advanced periductal fibrosis and bile duct cancer (cholangiocarcinoma). We investigated the relationship between plasma levels of Interleukin (IL)-6 and the risk of developing advanced fibrosis and bile duct cancer from chronic Opisthorchis infection. We show that IL-6 circulates in plasma at concentrations 58 times higher in individuals with advanced fibrosis than age, sex, and nearest-neighbor matched controls and 221 times higher in individuals with bile duct cancer than controls. We also observed a dose-response relationship between increasing levels of plasma IL-6 and increasing risk of advanced fibrosis and bile duct cancer; for example, in age and sex adjusted analyses, individuals with the highest quartiles of plasma IL-6 had a 19 times greater risk of developing advanced periductal fibrosis and a 150 times greater risk of developing of bile duct cancer than individuals with no detectable level of plasma IL-6. Finally, we show that a single plasma IL-6 measurement has excellent positive predictive value for the detection of both advanced bile duct fibrosis and bile duct cancer in regions with high *O. viverrini* transmission. These data support our hypothesis that common mechanisms drive bile duct fibrosis and bile duct tumorogenesis from chronic *O. viverrini* infection. Our study also adds a unique aspect to the literature on circulating levels of IL-6 as an immune marker of hepatobiliary pathology by showing that high levels of circulating IL-6 in plasma are not related to infection with *O. viverrini*, but to the development of the advanced and often lethal pathologies resulting from chronic *O. viverrini* infection.

## Author Summary

*O. viverrini* is among the few parasites considered a Class 1 carcinogen because of its strong association with bile duct cancer (cholangiocarcinoma). Currently, more than 40 million people are infected with *O. viverrini* worldwide. Thailand has the highest prevalence of *O. viverrini* at 10 million people infected and also the highest incidence of Opisthorchis-associated bile duct cancer (cholangiocarcinoma) in the world. In the current study, we also show that levels of IL-6 in plasma are associated in a dose-dependent fashion with Opisthorchis-induced bile duct fibrosis (periductal fibrosis) and cholangiocarcinoma. More importantly, we show that *O. viverrini* infection alone does not elevate IL-6 levels in the plasma. It is only in the presence of these advanced pathologies (advanced fibrosis or cholangiocarcinoma) from chronic *O. viverrini* infection that significantly elevates plasma levels of IL-6 are observed. Moreover, we show that plasma IL-6 is an easily accessible biomarker for the detection of advanced periductal fibrosis and cholangiocarcinoma, which would be a critical advance for this region of Thailand and other countries in Southeast Asia, where the prevalence of *O. viverrini* infection can reach as high as 80% and the incidence of bile duct cancer is the highest in the world.

## Introduction

Over 750 million people (10% of the human population) are at risk of infection with a food-borne trematode, with more than 40 million people currently infected [1]. *Opisthorchis viverrini* is considered among the most important of the food-borne trematodes due to its strong association with hepatobiliary pathologies that include advanced bile duct fibrosis (periductal fibrosis or APF) [2]–[5] and cholangiocarcinoma (CCA) [3], [6]–[11]. In Thailand, an estimated 10 million people are infected with *O. viverrini* [12], where uncooked cyprinoid fish (the intermediate host for the parasite) are a staple of the diet [13]. While infection with *O. viverrini* can be eliminated by chemotherapy (the anthelmintic praziquantel), culinary practices in Thailand result in rapid and prolonged re-infection after treatment. In the Northeastern region of Thailand (Isaan), individuals are often infected with *O. viverrini* for a lifetime [7], [14], [15].

As shown in our community-based ultrasound studies along the Chi River basin in Isaan (Khon Kaen, Thailand) [16], [17], important pathogenic changes occur early and asymptotically to the bile duct during *O. viverrini* infection, including fibrosis in the intrahepatic bile duct (periductal fibrosis). As with other forms of hepatic fibrosis, periductal fibrosis from chronic opisthorchiasis is probably the result of repeated injury sustained by the biliary epithelium from a combination of the mechanical, toxic, and immune mechanisms of the fluke in the bile duct [for review see [18]]. As individuals are infected with *O. viverrini* for many years (often a lifetime), a persistent cycle of tissue damage and repair takes place in the intrahepatic biliary ducts, creating a chronic inflammatory milieu that stimulates periductal fibrogenesis [for review of this process see [18], [19]]. In both the animal and human models of chronic *O. viverrini* infection, this fibrotic deposition along the biliary epithelium is a precursor event to CCA. For example, during the final phase of *O. viverrini* infection in the hamster model (at 12 weeks after infection), the inflamed biliary epithelium manifests fibrosis throughout its length, which is followed rapidly by tumorogenesis [20], [21]. In human autopsy studies, extensive fibrotic deposition in the intrahepatic bile duct routinely accompanies CCA tumors [10], [22], [23]. Although the exact mechanism has yet to be determined, advanced fibrotic lesions may precede tumorogenesis in the bile duct by producing a “smoldering and chronic inflammatory milieu” [24], [25]. A key factor in the maintenance of this chronic inflammatory milieu is the production of soluble growth factors such as the cytokine IL-6 [24], [25]. Studies of other hepatic pathogens also strongly implicate IL-6 in progressive pathogenic fibrosis and carcinogenesis, including hepatocellular fibrosis and hepatocellular carcinoma (HCC) from Hepatitis B (HBV) and Hepatitis C Virus (HCV) infection [26], [27].

Due to its role in systemic inflammation, IL-6 is readily detected in plasma [28]. As such, we sought to determine if the concentration of IL-6 in the plasma of *O. viverrini* infected individuals with APF and CCA was higher than age, sex, and nearest-neighbor matched controls infected with *O. viverrini* but without these advanced pathologies. We also sought to determine the sensitivity and specificity of a single measurement of plasma IL-6 to detect APF or CCA in these same individuals. Given the poor prognosis associated with CCA, especially in resource-poor settings such as Thailand, an early marker for the risk of the hepatobiliary pathologies related to *O. viverrini* infection is urgently needed.

## Materials and Methods

### Study design and setting

The current study presents baseline data collected from a community–based cohort study of the risk factors associated with the development of Advanced Periductal Fibrosis (APF) from chronic opisthorchiasis (Table 1). A detailed description of this population and the methods can be found in [17]. Individuals from seven villages with high *O. viverrini* transmission, including Nongnangkwan, Nongmuang, Loopka, Nongkham, Nongno, Lawa and Chikokor in the vicinity of Khon Kaen (Thailand) were surveyed. From this group of *O. viverrini*-infected individuals, 184 males and 236 females between the ages of 20 and 60 years (inclusive) were enrolled into the study as “cases” or age, sex, and nearest neighbor matched “controls”. Individuals were enrolled as “cases” on the basis of an ultrasound (US) in which APF was determined [17]. Individuals who were positive for *O. viverrini* but negative for APF by US were defined as “controls” and matched with cases by age (ten year age intervals), sex, and residence in the same village (nearest-neighbor method). A positive pregnancy test excluded female volunteers from participation in the ultrasonography and blood draw. As such, 210 individuals were identified as cases and matched 210 individuals as controls. Both cases and control were asked to provide 30 ml of blood for baseline immunology and hematological parameters. Individuals positive for *O. viverrini* were referred to the local public health outpost for treatment with praziquantel.

**Table 1:** Descriptive statistics for cases and controls used in the current study.

		Advanced Periductal Fibrosis		
Characteristics	Non-endemicControls^1^	NegativeControls^2^	PositiveCases^3^	CCACases^4^
	N (%)	N (%)	N (%)	N (%)
Total	21	210	210	121
Sex				
Male	11 (52.4%)	92 (43.8%)	92 (43.8%)	83 (68.6%)
Female	10 (47.6%)	118 (56.2%)	118 (56.2%)	38 (31.4%)
Age (in years)				
20–29	15 (71.4%)	6 (2.9%)	6 (2.9%)	0 (0.0%)
30–39	1 (4.8%)	40 (19.0%)	39 (18.6%)	6 (7.2%)
40–49	3 (14.3%)	86 (41.0%)	87 (41.4%)	20 (24.1%)
50+	2 (9.5%)	78 (37.1%)	78 (37.1%)	57 (68.7%)

1 Non-endemic control” refers to age-matched Thai individuals who have never resided in an area with *O. viverrini* transmission.

2 A control refers to an *O. viverrini*-infected individual who is age, sex, and nearest neighbor matched to a “case” but are negative for Advanced Periductal Fibrosis.

3 A case refers to *O. viverrini* infected individual who is positive for Advanced Periductal Fibrosis.

4 A case in this column refers to an individual with histologically proven *O. viverrini* associated cholangiocarcinoma (CCA) from the biological repository of the Liver Fluke and Cholangiocarcinoma Research Center, Faculty of Medicine, Khon Kaen University, Thailand.

All subjects provided written informed consent using Informed Consents Forms approved by the Ethics Committee of Khon Kaen University School of Medicine, Khon Kaen, Thailand (reference number HE480528) and the Institutional Review Board of the George Washington University School of Medicine, Washington, D.C (GWUMC IRB# 020864).

### CCA cases

Aliquots of plasma from 121 cases of histologically proven, *O. viverrini* associated CCA cases were pulled from the biological repository of the Liver Fluke and Cholangiocarcinoma Research Center, Faculty of Medicine, Khon Kaen University, Thailand (Table 1). Of these pulled samples, 83 were male and 38 were female. All samples were from patients who had liver resection surgery as a part of palliative care for *O. viverrini*-associated CCA at the Khon Kaen University Srinagarind Hospital, Khon Kaen, Thailand.

### Ultrasonography

A detailed description of the ultrasonography methods used in this study can be found in the following references [16], [17]. Briefly, a mobile, high-resolution ultrasound (US) machine (GE model LOGIQ Book XP) was used. Hepatobiliary abnormalities including portal vein radical echoes, echoes in liver parenchyma, indistinct gallbladder wall, gallbladder size, sludge and suspected CCA were graded and recorded as previously described [16], [17]. Individuals were classified as “Non-Advanced Periductal Fibrosis” or “controls” if the US grade was 0 or 1, and “Advanced Periductal Fibrosis” or “case” if the US grade was 2 or 3 as described in detail in our previous study [16], [17]. Individuals with alcoholic liver disease, which is seen as fatty liver by US exam, were excluded from the analysis component of the study. Also, individuals with marked hepatic fibrosis not related to *O. viverrini* infection (e.g., cirrhosis from HBV or HCV) were also excluded from the analysis component study.

### Fecal exams to determine *O. viverrini* infection status

Eggs/parasite identification and egg counts were performed by certified medical technicians using light microscopy at 10× and 40× magnifications as described in detail in our previous study [16].

### Plasma handling

Of the approximately 30 ml of blood collected from participants, 8 ml of blood were collected in heparinized tubes for the measurement of plasma cytokines. Plasma aliquots were grouped in case-control sets that were handled together throughout the processing and biochemical analysis. After blood draw, plasma was separated and immediately aliquoted into 500 microliter (mL) cryogenic tubes and frozen at −80°C until use. Biochemical analysis was done on all samples simultaneously, with the position of plasma samples varying at random during the processing. Plasma samples from CCA patients were taken at the time of liver resection, separated and aliquoted as above, and then stored at −80°C until use. CCA plasma samples were also randomly interspersed among the cases and control sample sets during biochemical analysis. The 21 non-endemic *O. viverrini* negative control plasma were handled as above and interspersed repeatedly at random among the actual case-control samples during biochemical analysis.

### Exploratory plasma cytokine measurements

Cytokine levels in plasma were examined for IL-1β, IL-2, IL-4, IL-5, IL-6, IL-8, IL-10, IL-12p70, interferon (INF) γ, tumor necrosis factor (TNFα), and TNFβ production. The quantification of plasma cytokines was analyzed using commercial FlowCytomix bead-based multiplexing assays kits (Beckman-Coulter). Values were quantified from standard curves using human recombinant cytokines. A quantile regression model was used to determine the median along with 95% confidence intervals (95% CI) to estimate the differences between cases and controls for plasma cytokine levels. When the data suggested a significant difference between cases and controls, such as the levels of plasma IL-6, the plasma were analyzed again using a sandwich ELISA as described below.

### IL-6 ELISA

IL-6 cytokine levels in the plasma were measured by sandwich enzyme-linked immunoadsorbent assay (ELISA) using a DuoSet (R & D systems, Inc) according to the manufacturer's instruction. The level of IL-6 was determined by interpolating the Optical Density of sample duplicates into a 4-parameter logistic-log model of a standard curve of recombinant human IL-6 run in serial dilutions on each ELISA plate. The concentration of IL-6 level is expressed as picograms per microliter.

### Statistics for Odd Ratios by quartile of IL-6 plasma concentration

The percent distribution of selected demographic characteristics were calculated for the four study groups: (1) non-endemic negative controls, (2) *O. viverrini* positive (OV+) and APF negative (controls), (3) OV+ APF positive (APF cases), and (4) OV+ and CCA+ (CCA cases). Box and whisker plots display the distribution of plasma IL-6 according to these groups. Age and sex adjusted Odds Ratios (OR) and 95% Confidence Intervals (CIs) for quartiles of plasma IL-6 concentration and their association with APF or CCA status were determined using age and sex adjusted multiple logistic regression analyses. A chi-square test for trend was also used to test the effect of increasing quartile level of plasma IL-6 on increasing risk of APF or CCA. The significance level for all tests was set at 0.05, with a Bonferroni correction for multiple testing. All statistical tests were two-sided. All analyses were performed using Stata version 10 (College Station, TX).

### Statistics for clinical epidemiology of IL-6 plasma concentration and APF and CCA

Receiver-operating-characteristic (ROC) curves were obtained by plotting the sensitivity versus 1–specificity for the full range of IL-6 cut-off points in picograms per milliliter (pg/mL) for both cases and controls to estimate the cutoff value that had the highest overall validity. “Sensitivity” was calculated as the number of individuals positive for APF or CCA testing positive for plasma IL-6 at various cutpoints (by pg/mL) divided by the total number of cases. “Specificity” was calculated as the number of individuals negative for APF and CCA (controls) testing negative for plasma IL-6 at various cutpoints (by pg/mL) divided by the total number of controls. The area under the ROC curve was calculated by determining the probability of correctly identifying (accuracy) a randomly selected participant as either a case (APF positive or CCA positive) or a non-case (APF negative or CCA negative). The 45-degree line in each ROC curve graph subsumes an area equal to 0.5 (50%), which is equivalent to using a coin toss procedure to classify participants as either cases or controls. Using the ROC curves, an optimal cutpoint was determined for the concentration of plasma IL-6 that maximized the sensitivity and specificity in classifying an individual at APF+ or CCA+. Based on this cutoff-point, the positive predictive value (PPV), and negative predictive value (NPV) of plasma IL-6 concentration to detected APF+ or CCA+ status were determined. All analyses were performed using Stata version 10 (College Station, TX).

## Results

### Study participants

As part of the inclusion criteria of the study, both cases and controls had to be positive for *O. viverrini* as determined by the presence of at least a single parasite ovum in feces. The baseline characteristics of the enrolled participants are shown in Table 1, with 210 cases (*O. viverrini*+ and APF+) and 210 controls (*O. viverrini*+ and APF−). The controls were matched with cases by age (ten year age intervals), sex, infection (*O. viverrini* positive) and nearest-neighbor status (same village). The study sample included more females (56.2%) than males (43.8%) (P<0.001). The mean ages for cases (46.6 years of age) and controls (46.6 years of age) did not differ significantly (P = 0.999). No statistically significant difference (P>0.811) was observed in the intensity of *O. viverrini* infection between cases (median = 142 epg) and controls (median = 130 epg). As shown in Table 1, both case and control groups had a similar distribution of individuals when age was stratified by ten-year intervals.

Among the 121 CCA samples in this study (Table 1), 117 (96.7%) were hepatectomies and 4 (3.3%) were small biopsy specimens. Of the 117 hepatectomies, 74 (63.2%) were of the mass-forming type and 8 (6.8%), 21 (18%), and 14 (12%) were periductal-infiltrating, invasive intraductal, and mixed types, respectively. Histologically, there were 41 well-differentiated (33.9%), 8 moderately differentiated (6.6%), 9 poorly differentiated adenocarcinomas (7.4%), 61 papillary carcinomas (50.4%), and 2 adenosquamous carcinomas (1.6%) of the 121 CCA cases studied.

### The concentration of plasma IL-6 is significantly elevated in individuals with Opisthorchis-induced APF and Opisthorchis-induced CCA

**Figure 1:**
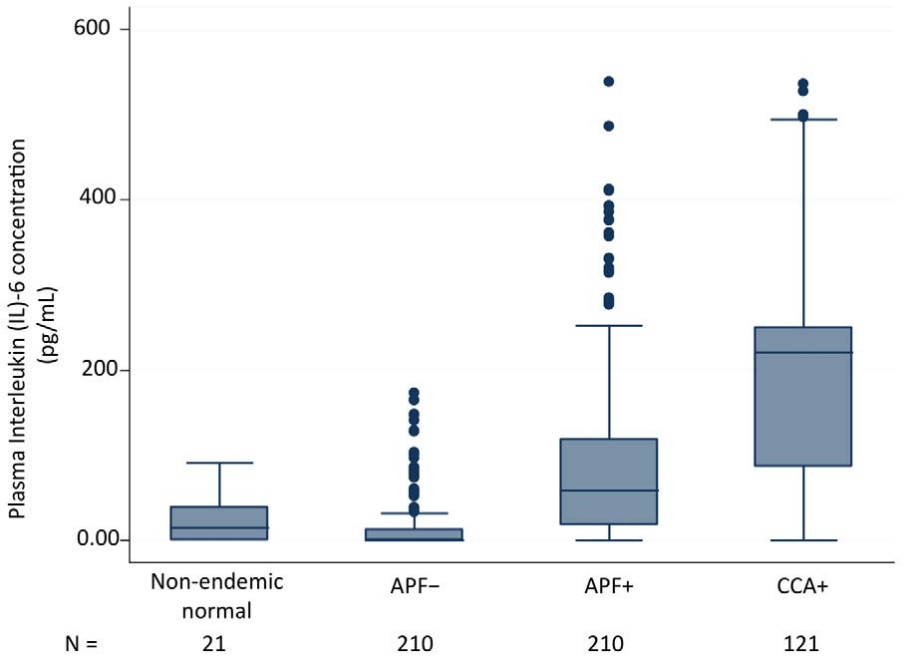
Plasma IL-6 levels are significantly elevated in individuals with Opisthorchis-induced APF and Opisthorchis-induced CCA. The distribution of plasma concentration of Interleukin (IL)-6) in picograms per milliliter is shown in boxplots by study group. The bottom and top of each box represent the 25th and 75th percentile (the lower and upper quartiles, respectively) of IL-6 concentration in plasma per group. The band near the middle of each box represents the median value of IL-6 concentration per group. The whiskers represent the minimum (bottom) and maximum (top) of IL-6 concentration per group.

Of the 11 cytokines tested only levels of the inflammatory cytokine IL-6 were significantly elevated in individuals with advanced periductal fibrosis and CCA compared to controls. The frequency distributions of plasma IL-6 concentration for all groups are presented in Figure 1. The IL-6 concentrations in plasma for both cases and controls ranged between <0.01 pg/mL (undetectable) and 538.6 pg/ml, with a much wider range in APF cases (<0.01 to 538.6 pg/ml) and CCA cases (<0.01 to 536.2 pg/ml) than APF- controls (<0.01 to 173.1 pg/ml). An even narrower range of plasma IL-6 concentration was recorded for the non-endemic controls (<0.01 to 91.4 pg/ml). These data indicate that there is far wider variation for the concentration of plasma IL-6 in the advanced forms of *O. viverrini* induced pathogenesis than in individuals with *O. viverrini* infection but no *O. viverrini* associated pathology. Moreover, the median levels of plasma IL-6 concentration were 58 times higher in APF+ cases than in controls (58 versus 1 pg/ml; P<0.001) and 221 times higher in CCA cases than in controls (221 versus 1 pg/ml; P<0.001). CCA cases also had higher levels of plasma IL-6 than APF cases (221 versus 58 pg/ml; P<0.001). Non-endemic controls had significantly lower (mostly undetectable or <0.01 pg/mL) plasma IL-6 concentrations (P<0.0001).

### Elevated levels of plasma IL-6 significantly increase the risk of APF in a dose-dependent manner

Increasing plasma concentrations of IL-6 significantly increased the risk of APF: for every 10 pg/ml increase of IL-6, there is an increase in the risk of APF by 22% (OR = 1.22; 95% CI 1.16 to 1.29, P<0.001) in a model adjusted for age and sex (data not shown). As shown in Table 2, the risk of APF increased with increasing quartile concentration of plasma IL-6: OR = 1.00 for Quartile 1 (reference quartile); OR = 3.94 (95% CI 2.21 to 6.81) for Quartile 2; OR = 7.95 (95% CI 4.73 to 13.36, P = <0.001) for Quartile 3; and OR = 18.94 (95%CI 10.17 to 35.25, P = <0.001) for Quartile 4. A strong and significant trend for increasing quartile concentration of IL-6 and increasing risk of APF was found compared to individuals with no detectable levels of plasma IL-6 (P<0.001).

**Table 2:** Odds Ratios for plasma IL-6 levels for *O. viverrini* infected individuals with and without Advanced Periductal Fibrosis.

	Quartile IL-6 (range in pg/ml)				
	1^1^	2	3	4	*P* for Trend
	(<0.01)	(0.01 to 21.62)	(21.63 to 82.79)	(82.80 to 358.63)	
*n*	180	76	128	127	
Crude					*P* = <0.001
OR^2^	1.00	3.88	8.02	18.55	
95% CI^3^	–	2.21–6.81	4.78–13.46	9.99–34.42	
*P*	–	<0.001	<0.001	<0.001	
Adjusted^4^					NA^5^
OR	1.00	3.94	7.95	18.94	
95% CI	–	2.24–6.95	4.73–13.36	10.17–35.25	
*P*	–	<0.001	<0.001	<0.001	

1 Individuals with undetectable levels of plasma IL-6 concentration are used as the reference group in Quartile 1.

2 Odds Ratio.

3 95% Confidence Interval.

4 Models were adjusted for age and sex simultaneously.

5 Not Available.

### Elevated levels of plasma IL-6 significantly increase the risk of developing CCA in a dose-dependent manner

Elevated plasma concentrations of IL-6 significantly increased the risk of CCA: for every 10 pg/ml increase of plasma IL-6 concentration, there was a 26% (OR = 1.26; 95% CI 1.19 to 1.34, P<0.001) increase in the risk of CCA in a model adjusted for age and sex. Table 3 shows that the risk of CCA increased in the 3rd and 4th highest quartile concentrations of plasma IL-6: OR = 4.55 (95% CI 2.05 to 10.11, P = <0.001) for Quartile 3 and OR = 149.11 (95% CI 40.42 to 550.15, P = <0.001) for Quartile 4. A strong and significant trend (P<0.001) for increasing quartile concentration of IL-6 and increasing risk of CCA was also found compared to individuals with no detectable level of plasma IL-6 (<0.01 pg/mL). Note that the term “control” refers to the age, sex, and nearest-neighbor matched controls for APF group and not for the CCA group and that the logistic model is adjusted for age and sex.

**Table 3:** Odds Ratios for plasma IL-6 levels for *O. viverrini* infected individuals with^1^ and without Cholangiocarcinoma (CCA)^2^.

	Quartile IL-6 (range in pg/ml)				
	1^3^	2	3	4	*P* for Trend
	(<0.01)	(0.01 to 21.62)	(21.63 to 82.79)	(82.80 to 358.64)	
N	145	21	83	82	
Crude					
OR^4^	1.00	0.47	4.76	182.46	*P* = <0.001
95% CI^5^	–	0.06–3.75	2/33–9.73	58.01–573.96	
*P*	–	0.475	<0.001	<0.001	
Adjusted^6^					
OR	1.00	0.21	4.55	149.11	NA^7^
95% CI	–	0.02–2.30	2.05–10.11	40.42–550.15	

1 Individual without CCA are the controls from the case-control study in Table 2, that is, *O. viverrini* infected individuals who are negative for Advanced Periductal Fibrosis (APF) by ultrasound and were age, sex, and nearest-neighbor matched with APF positive individuals (see Table 1).

2 CCA cases were from the biological repository of the Liver Fluke and Cholangiocarcinoma Research Center, Faculty of Medicine, Khon Kaen University, Thailand.

3 Individuals with undetectable levels of plasma IL-6 concentration are used as the reference group in Quartile 1.

4 Odds Ratio.

5 95% Confidence Interval.

6 Models were adjusted for age and sex simultaneously.

7 Not Available.

### Plasma IL-6 concentrations can be used to detect individuals with Opisthorchis-induced advanced periductal fibrosis (APF)

**Figure 2:**
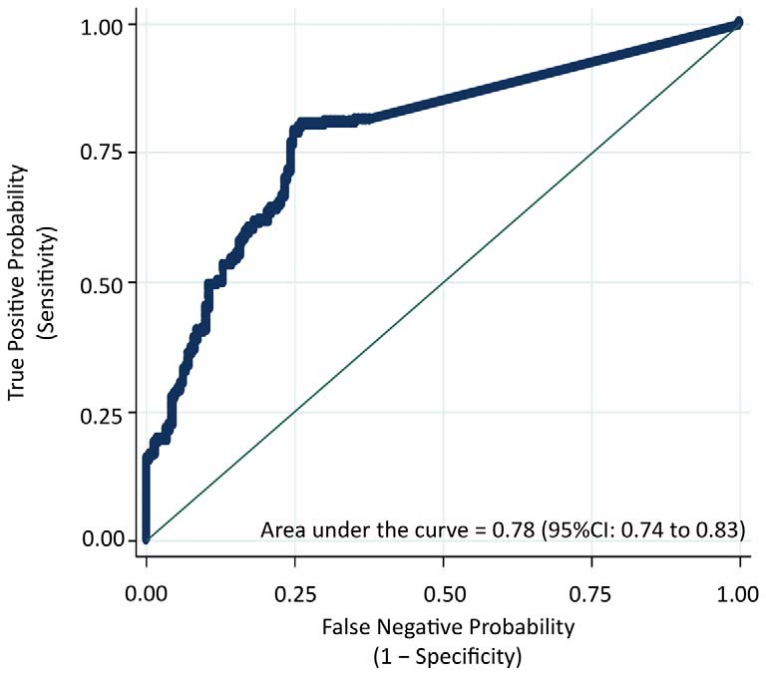
Plasma IL-6 concentrations can be used to detect individuals with Opisthorchis-induced advanced periductal fibrosis. A receiver-operating-characteristic (ROC) curve plots the True Positive Probability (sensitivity) against the False Negative Probability (1– specificity) for the full range of IL-6 cut-off points for the detection of Opisthorchis-induced Advanced Periductal Fibrosis (APF) as determined by ultrasound. The area under the ROC curve is interpreted as the probability of correctly identifying (accuracy) a randomly selected participant as either a case (APF positive) or a non-case (APF negative). The 45-degree line in the graph subsumes an area equal to 0.50 (50%), which is equivalent to using a coin toss procedure to classify participants as either cases or controls. As determined by this ROC curve, the optimal cutpoint is 11 pg/mL of plasma IL-6, which refers to the concentration that maximizes the “sensitivity” and the “specificity” of classifying an individual at APF positive. Based on this cutoff point, the Positive Predictive Value (PPV) and the Negative Predictive Value (NPV) of plasma IL-6 concentration to detect APF was also determined (see Table 4). All analyses were performed using Stata version 10 (College Station, TX). The ROC was derived from 210 *O. viverrini* infected individuals with Advanced Periductal Fibrosis as determined by ultrasound (gold standard) versus 210 age, sex, and nearest neighbor-matched controls (*O. viverrini* infected but negative for APF.)

Figure 2 shows the ROC curve obtained by plotting the True Positive Probability (sensitivity) against the False Negative Probability (1–specificity) for the entire range of IL-6 cut-off points to predict the presence of APF. Using a cutoff of greater than 11 pg/mL of plasma IL-6, the sensitivity for the detection of APF was 80% and the specificity was 74% (Table 4). In addition, for the cutoff of greater than 11 pg/mL of plasma IL-6, the area under the ROC curve, which is an indication of the “accuracy” of the test or proportion of all tests that have given the correct result, was 78% (95%CI 74% to 83%). Table 4 also shows the Positive Predictive Value (PPV) and Negative Predictive Value (NPV) of 76% and 79%, respectively, for the detection of APF using plasma IL-6 levels greater than 11 pg/mL.

**Table 4:** The diagnostic utility of plasma Interleukin (IL)-6 levels for Opisthorchis-induced pathologies.

	N			Cut off	Percent (%)				
	Case	Con^1^	Tot	>pg/mL	Sen^2^	Spec^3^	Acc^4^	PPV^5^	NPV^6^
APF^7^	210	210	420	11	80	74	77	76	79
CCA^8^	121	210	331	64	80	90	86	82	88

1 Age, sex, and nearest-neighbor matched individuals, who were positive for *O. viverrini* infection and negative for APF as determined by ultrasound (US) were included in the analyses and shown as controls in Table 1.

2 Sensitivity.

3 Specificity.

**Figure 3:**
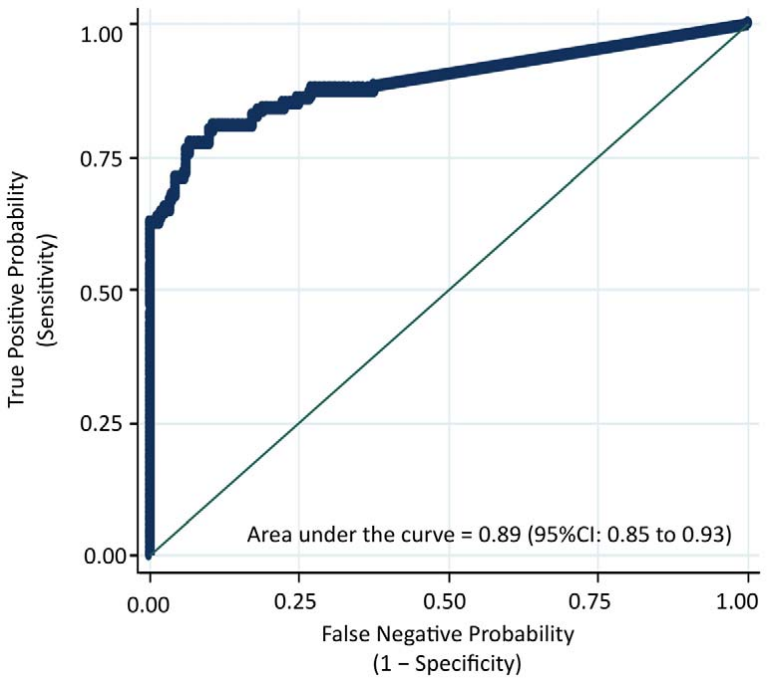
Plasma IL-6 concentrations can be used to detect O. viverrini infected individuals with cholangiocarcinoma (CCA). A receiver-operating-characteristic (ROC) curve plots the True Positive Probability (sensitivity) against the False Negative Probability (1– specificity) for the full range of IL-6 cut-off points for the determination of Opisthorchis-induced cholangiocarcinoma (CCA). The area under the ROC curve is interpreted as the probability of correctly identifying (accuracy) a randomly selected participant as either a case (APF positive) or a non-case (APF negative). The 45-degree line in the graph subsumes an area equal to 0.50 (50%), which is equivalent to using a coin toss procedure to classify participants as either cases or controls. As determined by this ROC curve, the optimal cutpoint is 11 pg/mL of plasma IL-6, which refers to the concentration that maximizes the “sensitivity” and the “specificity” of classifying an individual at APF positive. Based on this cutoff point, the Positive Predictive Value (PPV) and the Negative Predictive Value (NPV) of plasma IL-6 concentration to detect APF was also determined (see Table 4). All analyses were performed using Stata version 10 (College Station, TX). The ROC was derived from 121 cases of histologically proven, *O. viverrini* associated CCA cases from the biological repository of the Liver Fluke and Cholangiocarcinoma Research Center, Faculty of Medicine, Khon Kaen University, Thailand and 210 individuals who are *O. viverrini* infected but negative for APF and CCA.

4 Accuracy of the test or the area under the Receiver-Operating Characteristic (ROC) curves in Figures 2 and 3.

5 Positive Predictive Value.

6 Negative Predictive Value.

7 *O. viverrini* infected individuals with Advanced Periductal Fibrosis as determined by US and shown as cases in Table 1.

8 Plasma samples from histologically confirmed Opisthorchis-induced cholangiocarcinoma from the Liver Fluke and Cholangiocarcinoma Research Center, Faculty of Medicine, Khon Kaen University, Thailand.

### Plasma IL-6 concentrations can be used to detect *O. viverrini* infected individuals with cholangiocarcinoma (CCA)

Figure 3 shows the ROC curve obtained by plotting the True Positive Probability (sensitivity) against the False Negative Probability (1–specificity) for the entire range of IL-6 cut-off points to predict the presence of CCA. Using a cutoff of greater than 64 pg/mL of plasma IL-6, the sensitivity for the detection of CCA was 80% and the specificity was 90% (Table 4). In addition, for the optimal cutoff of >64 pg/mL of plasma IL-6, the area under the ROC curve, which is an indication of the “accuracy” of the test, was 89% (95% CI = 85% to 93%). Table 4 also shows the Positive Predictive Value (PPV) and Negative Predictive Value (NPV) of 82% and 88%, respectively, for the detection of APF using plasma IL-6 levels >64 pg/mL. Note that the term control refers to the age, sex, and nearest neighbor-matched controls for APF group and not for the CCA group.

## Discussion

These data show that elevated plasma concentrations of IL-6 are associated with a marked and significant increase in the risk of *O. viverrini*-associated APF and CCA. As shown in Tables 2 and 3, increasing levels of plasma IL-6 associate with increasing risk of these advanced pathologies in a dose-dependent manner: for example, individuals with the highest quartiles of plasma IL-6 concentration had a 19 times greater risk of developing APF and a 150 times greater risk of developing CCA than individuals with undetectable levels of plasma IL-6 (<0.01 g/mL). As the data were collected by a cross-sectional study design, the complicity of IL-6 in these pathogenic processes remains to be determined: that is, elevated plasma concentrations of IL-6 may simply reflect the presence of these hepatobiliary abnormalities or, as shown in a number of other studies, elevated plasma concentrations of IL-6 may play a key role in these pathogenic processes by creating an inflammatory milieu that favors fibrotic deposition and carcinogenesis in the bile duct [for reviews see [28]–[30]]. Notably, our data show that *O. viverrini* infection alone does not elevate IL-6 levels circulating in the plasma: e.g., age and sex matched *O. viverrini* infected individuals without APF or CCA had negligible levels of IL-6 in their plasma. It is only in the presence of advanced pathology from chronic opisthorchiasis that plasma concentrations of IL-6 are significantly elevated.

This study also shows that plasma IL-6 concentration can be used to detect the risk for the advanced pathologies associated with *O. viverrini* infection, many of which are subclinical. Our data on the predictive values of a single measurement of plasma IL-6 to detect CCA closely resemble recent data from Cheon and co-workers [31] as well as other studies that have also reported a high sensitivity and specificity for IL-6 in serum for non-Opisthorchis-associated CCA [see [32] for review]. Our study adds a unique aspect to the literature on circulating levels of IL-6 as an immune marker of hepatobiliary pathology by showing that high levels of circulating IL-6 in plasma are not related to infection with *O. viverrini*, but to the development of the advanced and often lethal pathologies resulting from chronic *O. viverrini* infection: i.e., age and sex matched controls with *O. viverrini* infection and no pathology (APF- and CCA-) had undetectable levels of this inflammatory cytokine in their plasma. Moreover, the ability of a single IL-6 measurement to detect risk for *O. viverrini* associated CCA and *O. viverrini* associated pathogenic fibrosis in the bile duct (APF) is especially important in regions where *O. viverrini* is endemic. In Isaan, Thailand, for example, the prevalence of *O. viverrini* infection can reach as high as 79%; hence, an easily accessible immune marker which can distinguish between infection with *O. viverrini* and the advanced pathology induced by this parasite would be particularly useful, where the incidence of intraheptic CCA is among the highest in the world [33]. Currently, Thai individuals in *O. viverrini* endemic areas are diagnosed only in the most advanced stages of CCA, when treatment is essentially palliative. An easily measurable biomarker for a precursor stage to CCA such as APF would be of great public health importance by identifying those at greatest risk of CCA.

IL-6 is produced by numerous cell types, with a broad range of effects, including the promotion of the innate immune response to pathogens as well as the subsequent chronic inflammatory reactions [28], [30], [34]. Once known as “hepatocyte-stimulating factor” (HSF), IL-6 has been shown to play a key role in chronic inflammatory conditions of the liver that lead to fibrotic lesions, e.g., chronic alcohol consumption or viral hepatitis [26], [35]. Our previous studies have shown that crude antigens extracts antigens from this parasite stimulate host PBMC from *O. viverrini* infected individuals to produce high levels of IL-6 [16]. Our new data indicate that elevated levels of IL-6 are not confined to the immune response to parasite antigens, but circulate at elevated levels in the plasma of individuals with advanced pathologies associated with chronic *O. viverrini* infection, a role consistent with the reputation of IL-6 as a key player in systemic inflammation. We speculate that elevated concentrations of IL-6 circulating in the plasma reflect the constant tissue repair response of the biliary epithelia to the chronic mechanical, toxic, and immune injury induced by the fluke [18]. When these tissue repair mechanisms are activated transiently, the normal hepatobiliary structure and function rapidly recovers: i.e., cholangiocytes would regenerate and replace the necrotic or apoptotic cells, a process associated with a local and transitory IL-6 response. However, as with chronic alcohol consumption or infection with either HBC or HCV infection, chronic opisthorchiasis creates a persistent inflammatory milieu that stimulates fibrotic deposition [25], [29], [36], a process that could be reflected or even partially induced by the elevated concentrations of IL-6 circulating in the plasma.

As with other inflammation associated hepatic pathologies [25], [29], [36], [37], tumors in the biliary tract may have activated the wound-healing programs of the host chronically infected with *O. viverrini* in an exaggerated and prolonged manner. A parallel scenario is the presence of pathogenic fibrosis before the development of HCC in patients with chronic HCV [for review see [27]]. In the case of HCC, it is hypothesized that the development of fibrosis and/or cirrhosis, plus a microenvironment conducive to genomic instability, promotes neoplastic transformation in areas of hepatic fibrosis. Prominent among these processes is the production of soluble growth factors such as IL-6 [25], [29], [36]. In the hamster animal model of *O. viverrini*-induced CCA, the constant injury to bile duct epithelium by the liver fluke engages a similar process [11]. During the early stage of infection (4 weeks), fluke injury to the biliary epithelium results in inflammatory cell infiltration, hyperplasia, and in the adenomatous change in the bile duct epithelium. By the chronic stage of the infection (12 weeks), the immune response has transformed into prolonged inflammation with fibrotic deposition along the bile duct wall and ultimately CCA [18], [38]–[41]. In autopsy studies [22], [23] and studies on liver tissue resected from Opisthorchis-induced CCA patients for palliative care [5], [18], fibrosis is routinely detected proximal to neoplastic bile duct tissue.

In summary, this study identifies a significant relationship between plasma IL-6 concentration and the advanced pathologies associated with chronic *O. viverrini* infection. While in other settings (e.g. Western countries) the causative agent of CCA remains obscure, the single most important risk factor for intrahepatic CCA in Thailand has long been established–infection with the liver fluke *O. viverrini* [9]. As such, an easily accessible biomarker such as plasma IL-6 would have great utility in predicting those at risk (APF) or already with early CCA in a setting, where half of the population are routinely exposed to this class I carcinogen *O. viverrini* through the daily consumption of raw fish [42].
